# The Algorithmic Paradox: How Artificial Intelligence Challenges the Traditional Framework of Clinical Practice Guidelines

**DOI:** 10.1002/gin2.70031

**Published:** 2025-07-16

**Authors:** Laiba Husain

**Affiliations:** ^1^ Department of Social and Behavioral Sciences, Peter O'Donnell Jr. School of Public Health University of Texas Southwestern Medical Center Dallas Texas USA

## 1 Introduction

The intersection of artificial intelligence and clinical practice guidelines represents a complex methodological challenge facing contemporary healthcare. Although over 1000 FDA‐approved artificial intelligence devices now operate within clinical settings [1], their integration with established guideline frameworks presents significant methodological and practical challenges. This convergence raises fundamental questions about evidence generation, clinical decision‐making authority and patient safety considerations.

AI healthcare applications have grown exponentially, with research publications increasing by 10.4% annually over 3 years, totalling 28,180 articles in 2024 [1]. However, only 19% of AI clinical trials published after 2021 cited CONSORT‐AI guidelines [2], revealing gaps between AI development and clinical reporting standards.

Traditional guidelines derive authority from systematic reviews of population‐based studies, providing standardised recommendations for consistent care [3]. AI systems generate individualised predictions through pattern recognition from large datasets, often diverging from population‐based guidelines. The challenge involves determining how these approaches can coexist within coherent clinical frameworks.

The FUTURE‐AI consensus guideline, developed by 117 experts across 50 countries, emphasises six principles—fairness, universality, traceability, usability, robustness and explainability—for integration within existing clinical governance structures [4].

## 2 Evidence Generation and Validation Challenges

The reproducibility challenges inherent in AI research present additional complications. General textual descriptions often lack sufficient detail about preprocessing, model training and validation procedures [5], making it difficult to assess the quality and reliability of AI‐generated evidence. This contrasts sharply with the transparency requirements typically expected in traditional clinical research that informs guideline development.

Furthermore, the dynamic nature of AI systems presents unique challenges for guideline developers. Unlike pharmaceutical interventions that remain consistent across implementations, AI systems may evolve through continuous learning algorithms, potentially altering their performance characteristics over time [6]. This temporal variability challenges the traditional assumption that evidence supporting guideline recommendations remains stable throughout the guideline's lifecycle, raising questions about how to maintain evidence currency in rapidly evolving technological environments.

The movement toward personalised medicine introduces additional complexity to the relationship between AI and clinical guidelines. The International Consortium for Personalised Medicine envisions healthcare transformation by 2030 through individualised treatment approaches that integrate genetic, lifestyle and environmental factors [7]. Although this vision holds promise for improving patient outcomes, it fundamentally challenges the epistemological foundation of clinical practice guidelines, which traditionally derive authority from population‐level evidence rather than individual‐level predictions.

Recent research in oncology demonstrates both the potential and the limitations of this tension. Studies indicate that biomarker‐guided personalised medicine can significantly improve outcomes for patients with specific genetic mutations, yet the broader applicability of such approaches across diverse patient populations remains unclear [7]. The challenge for guideline developers lies in determining when individual‐level predictions should supersede population‐based recommendations and establishing criteria for making such determinations safely and consistently.

The implementation challenges become more complex when considering that healthcare systems must accommodate both traditional guideline‐based care and emerging AI‐driven approaches. This dual requirement raises questions about resource allocation, training requirements and quality assurance mechanisms that current implementation science literature has not adequately addressed.

## 3 Regulatory and Governance Considerations

The governance implications of AI‐guideline integration extend beyond technical considerations to encompass professional liability, quality assurance and regulatory oversight. The guidance principles developed by the Guidelines International Network emphasise the need for systematic approaches to AI integration in guideline enterprises [3]. However, the relationship between regulatory approval of AI systems and their integration into clinical practice guidelines remains poorly defined.

Current regulatory frameworks focus primarily on device safety and efficacy rather than integration with clinical decision‐making protocols. Although regulatory bodies may approve AI diagnostic tools, the mechanisms by which such approvals translate into guideline recommendations for clinical use remain unclear. This gap creates potential inconsistencies between regulatory approval and clinical implementation guidance.

The governance challenges are compounded by questions about professional liability when AI recommendations conflict with established guidelines. Healthcare providers must navigate complex decisions about when to follow traditional guidelines versus AI‐generated recommendations, often without clear institutional policies or professional guidance to inform these choices.

## 4 Methodological Considerations for the Future

The healthcare community faces the challenge of developing frameworks that can accommodate both the rigour of traditional evidence‐based medicine and the potential benefits of AI‐driven clinical decision support. This may require fundamental reconsiderations of how clinical evidence is generated, evaluated and translated into practice recommendations.

One potential approach involves developing hybrid frameworks that incorporate both population‐based evidence and individual‐level predictions while maintaining clear criteria for when each approach is most appropriate. Such frameworks would need to address questions of evidence hierarchy, validation requirements and safety monitoring that current methodologies do not adequately encompass.

The development of such frameworks will require unprecedented collaboration between traditional guideline developers, AI researchers, regulatory bodies and clinical implementers. The challenge lies not merely in technical integration but in reconciling fundamentally different approaches to evidence generation and clinical decision‐making that have emerged from distinct intellectual and methodological traditions.

## 5 Implications for Clinical Practice

The integration of AI with clinical practice guidelines will likely require significant changes in how clinicians are trained, how healthcare institutions develop policies and how professional organisations establish standards of care. These changes must balance the potential benefits of technological innovation with the proven value of evidence‐based clinical protocols.

The resolution of these challenges will likely determine the trajectory of evidence‐based medicine in the coming decades and shape the relationship between human clinical judgement and algorithmic decision support in patient care. Success will require careful attention to both the opportunities and limitations of each approach, ensuring that technological advancement serves to enhance rather than replace the fundamental principles of safe, effective and equitable healthcare delivery.

The intersection of artificial intelligence and clinical practice guidelines represents both an opportunity and a challenge for modern healthcare. Although AI technologies offer potential benefits for improving clinical decision‐making and personalising patient care, their integration with established guideline frameworks requires careful consideration of evidence standards, safety requirements and governance structures. The healthcare community must navigate these complexities thoughtfully, ensuring that innovation enhances rather than compromises the quality and safety of patient care.

## Conflicts of Interest

The author declares no conflicts of interest.

## Data Availability

No datasets were generated or analysed during the preparation of this editorial commentary.
